# Contralateral spreading of substances following intratympanic nanoparticle-conjugated gentamicin injection in a rat model

**DOI:** 10.1038/s41598-020-75725-y

**Published:** 2020-10-29

**Authors:** Sang-Yeon Lee, Jeonghyo Kim, Sangjin Oh, Gaon Jung, Ki-Jae Jeong, Van Tan Tran, Dajeong Hwang, SungIl Kim, Jae-Jin Song, Myung-Whan Suh, Jaebeom Lee, Ja-Won Koo

**Affiliations:** 1 Department of Otorhinolaryngology-Head and Neck Surgery, Seoul National University Bundang Hospital, Seoul National University College of Medicine, 82, Gumi-ro 173, Bundang-gu, Seongnam-si, Gyeonggi-do 463-707 South Korea; 2 Department of Chemistry and Department of Chemical Enginnering and Applied Chemistry, Chungnam National University, Daejon, 34134 Republic of Korea; 3 Department of Cogno-Mechatronics Engineering, Busan National University, Busan, 46241 Republic of Korea; 4 Faculty of Biotechnology, Chemistry and Environmental Engineering, Phenikaa University, Hanoi, 10000 Vietnam; 5 AMO LIFE SCIENCE Co., Ltd., Seoul, 06527 Republic of Korea; 6 Department of Otorhinolaryngology-Head and Neck Surgery, Seoul National University Hospital, Seoul National University College of Medicine, Seoul, South Korea

**Keywords:** Medical research, Nanoscience and technology

## Abstract

This study was performed to investigate the Eustachian tube as a potential route for contralateral spreading following intratympanic nanoparticle (NP)-conjugated gentamicin injection in a rat model. Sprague–Dawley rats were divided into three groups and substances were injected in the right ear: group 1 (fluorescent magnetic nanoparticles [F-MNPs], *n* = 4), group 2 (F-MNP-conjugated gentamicin [F-MNP@GM], *n* = 2), and control group (no injections, *n* = 2). T2-weighted sequences corresponding to the regions of interest at 1, 2, and 3 h after intratympanic injection were evaluated, along with immunostaining fluorescence of both side cochlea. The heterogeneous signal intensity of F-MNPs and F-MNP@GM on T2-weighted images, observed in the ipsilateral tympanum, was also detected in the contralateral tympanum in 4 out of 6 rats, recapitulating fluorescent nanoparticles in the contralateral cochlear hair cells. Computational simulations demonstrate the contralateral spreading of particles by gravity force following intratympanic injection in a rat model. The diffusion rate of the contralateral spreading relies on the sizes and surface charges of particles. Collectively, the Eustachian tube could be a route for contralateral spreading following intratympanic injection. Caution should be taken when using the contralateral ear as a control study investigating inner-ear drug delivery through the transtympanic approach.

## Introduction

Meniere’s disease is a complex clinical entity that is characterized by episodic attacks of vertigo, fluctuating sensorineural hearing loss, aural fullness, and tinnitus^1^. Unpredictable attacks of vertigo are associated with high levels of disability in patients with Meniere’s disease^2^. Given the multifactorial nature of Meniere’s disease, stepwise treatment is generally performed to control the associated attacks of vertigo and preserve residual hearing. Although attacks of vertigo in the initial phase of the disease are controlled with conservative treatments, injections of gentamicin through the transtympanic approach are an effective treatment for attacks of vertigo in refractory Meniere’s disease^3,4^. The effectiveness of transtympanic gentamicin therapy is based on the kinetics of gentamicin uptake in the inner ear and the dynamic distribution of ototoxic gentamicin in the inner ear structures^5^. A previous study using an animal model indicated that gentamicin may cause deterioration of all cochlear cells following local administration to the round window^6^.

However, intratympanic gentamicin injection does not necessarily result in ototoxicity, as evident in reported intractable cases following gentamicin administration^4^. Individual differences in the permeability of the round window and the degree of endolymphatic hydrops are associated with the diffusion-to-clearance ratio of intratympanically injected substances^7,8^. Although gentamicin is mostly delivered to the basal turn of the cochlea via the round window, the Eustachian tube in the middle ear is a potential route for the clearance of gentamicin. A recent study found the diffuse staining of fluorescent material in the Eustachian tube following intratympanic injection^9^. Interestingly, we observed the temporal and spatial distributions of nanoparticle (NP)-conjugated gentamicin even in the contralateral middle and inner ear following intratympanic injection in a rat model, which led us to hypothesize that the Eustachian tube is a potential route for contralateral spreading. To the best of our knowledge, no studies have investigated the Eustachian tube as a potential route, although previous studies have suggested the possibility of contralateral spreading following intratympanic injection through the connection between the perilymph and cerebrospinal fluid from the cochlear aqueduct^5^.

Here, we report a previously undescribed potential route, the Eustachian tube, for contralateral spreading following intratympanic NP-conjugated gentamicin injection in a rat model, and discuss the possible underlying mechanism using computational simulations complimented by Multiphysics. The results of our study provide crucial insight into the contraindication of the contralateral ear as a control ear, especially in rat studies investigating inner ear delivery through the transtympanic approach.

## Results

### Characterization of F-MNP-conjugated gentamicin

**Figure 1:**
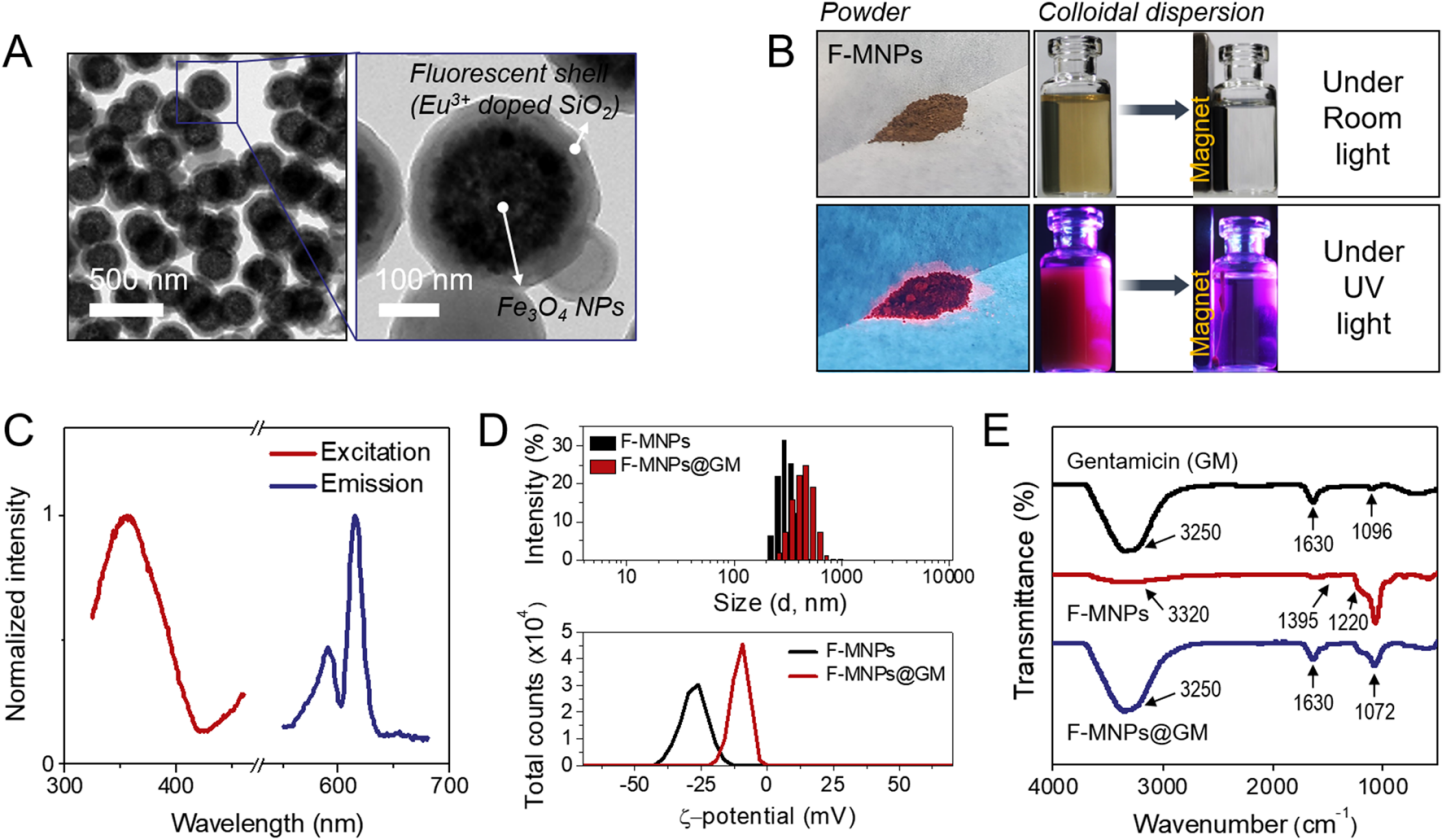
Characterization of fluorescent magnetic nanoparticles (F-MNPs) and F-MNP-conjugated gentamicin (F-MNP@GM). (**A**) Transmission electron microscopy (TEM) images of F-MNPs at different magnifications. (**B**) Photographs of the particle powder and dispersion under visible and UV light. (**C**) Excitation and emission spectra of F-MNPs. (**D**) Hydrodynamic diameter, zeta potential, and (**E**) Fourier transform infrared (FT-IR) spectrum of F-MNPs during the process of drug loading.

Multifunctional NPs that simultaneously exhibit fluorescence and magnetic properties would be beneficial as drug tracers in vivo. Fluorescent magnetite nanoparticles (F-MNPs) were fabricated by coating a silica (SiO_2_) shell onto MNPs with europium (III) (Eu^3+^) ions using a sol–gel method. Transmission electron microscopy (TEM) images showed that the resulting core–shell NPs had a uniform size of ~ 320 nm, in which the Eu-doped SiO_2_ shell layer could be distinguished from the core due to its low contrast in TEM (Fig. 1A). The hydrodynamic diameter of core magnetite NPs in DLS was 265.2 ± 2.3 nm, which increased to 338.5 ± 4.7 nm after coating with the fluorescent shell, indicating that the SiO_2_ shell has a thickness of approximately 36.7 nm (Supplementary Fig. S1A,B). This SiO_2_ layer acts as a spacer between the Eu ion and the magnetite particle surface, effectively avoiding fluorescence quenching by the iron oxide surfaces. Powder and dispersion of the particle appeared brown in visible light, but bright-red emission was observed under UV irradiation (Fig. 1B). In addition, because of its high magnetic responsiveness, the movement of F-MNPs could be easily controlled by an applied magnetic field. Pictures of F-MNP separation or 1D nanochain alignment along an external magnetic field under UV light provided direct evidence of the magnetic and fluorescent properties of the particles (Supplementary Fig. S1C). The photoluminescence (PL) spectra of F-MNPs are shown in Fig. 1C. The particles showed strong excitation and emission peaks at 355 nm and 615 nm, respectively; both were close to those of the pure Eu chelate, suggesting that Eu^3+^ was successfully embedded into the silica shell.

The gentamicin sulfate molecules were covalently bound to the F-MNPs based on the EDC/NHS coupling reaction, namely F-MNP-conjugated gentamicin (F-MNP@GM). The negative surface charge of the F-MNPs was reduced from − 22.5 ± 0.7 to − 7.2 ± 0.5 mV after gentamicin sulfate conjugation, which was attributed to the strong coordination of positively charged drug molecules on the NP surfaces (Fig. 1D)^10^. Fourier-transform infrared (FT-IR) spectroscopy showed further evidence of the formation of NP-conjugated gentamicin, as shown in Fig. 1E, where both characteristic bands from gentamicin and F-MNPs were observed. In the spectrum of gentamicin, the peak at 1630 cm^−1^ was related to the N–H group bending, and the band at 3250 cm^−1^ was attributed to –NH_2_ bond stretching. A band of C–O stretching vibration was observed at 1096 cm^−1^^11,12^. Carboxylic and SiO_2_ groups on the surface of F-MNPs were identified on the FT-IR spectra. The strong band at 1072 cm^−1^ corresponded to the Si–O–Si band of (3-aminopropyl) triethoxysilane (APTES)^13^. A band at 1220 cm^−1^ was ascribed to the formation of the C–N bond. The C–H vibration band appeared at 1395 cm^−1^, and the broad absorption band at 3320 cm^−1^ was attributed to O–H stretching vibration or absorbed water^14^. Although the peak at 1096 cm^−1^ was overlapped with the peak of the Si–O–Si band, the spectrum of F-MNPs@GM showed characteristic bands of gentamicin at 1630 and 3250 cm^−1^, which indicate that gentamicin sulfate was successfully loaded onto the surface of F-MNPs.

### MRI: visualization of intratympanically injected materials with time

A total of eight Sprague–Dawley rats (weight range 279–336 g) were used in the study and divided into three groups based on the injection materials and laterality: group 1, F-MNPs in the right ear and no injection in the left ear (*n* = 4); group 2, F-MNP@GM in the right ear and no injection in the left ear (*n* = 2); and group 3, control group, with no injection in either ear (*n* = 2) (Table 1).

**Table 1:** Three experimental groups of a total of eight Sprague–Dawley rats (weight range 279–336 g) divided according to the injection materials and laterality.

Group	Laterality	Materials	Conjugation	Approach	Dose (cc)	NP concentration (mg/mL), (injected volume [μL])
**Group 1**						
Rat (*n* = 4)	Rt	F-MNPs (COOH terminal)	NA	Intratympanic injection	0.05	1 mg/mL (700 μL)
	Lt	NO injection				
**Group 2**						
Rat (*n* = 2)	Rt	F-MNP@GM (COOH terminal)	Covalent binding (EDC/NHS)	Intratympanic injection	0.05–0.06	5 mg/mL (500 μL)
	Lt	NO injection				
**Group 3**						
Rat (*n* = 2)	Rt	NO injection	NA			
	Lt	NO injection				

*F-MNPs* fluorescent magnetic nanoparticles, *F-MNP@GM* F-MNP-conjugated gentamicin, *Rt* right, *Lt* left, *NA* not available.

**Figure 2:**
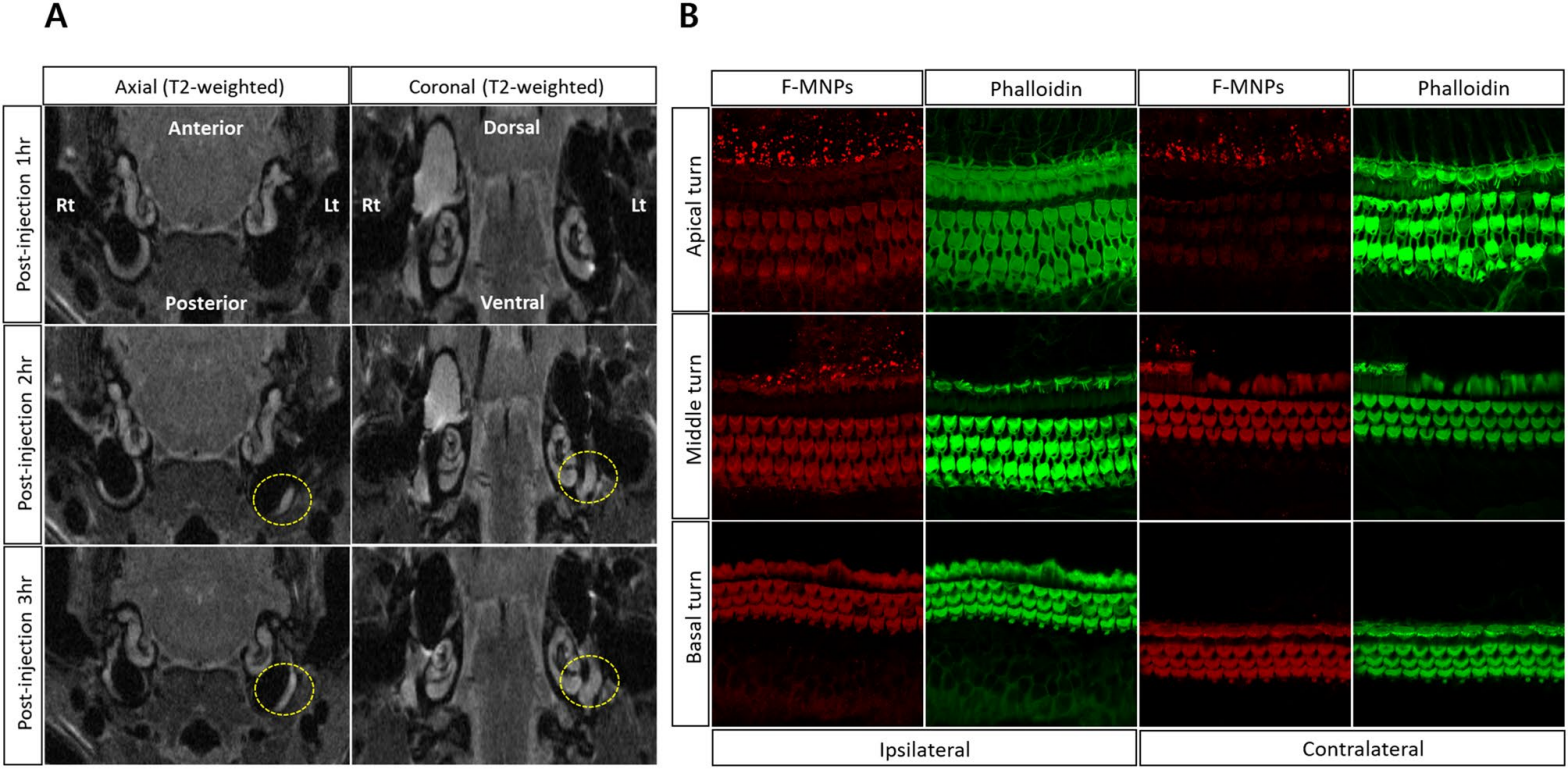
(**A**) Temporal visualization of intratympanically injected fluorescent magnetic nanoparticles (F-MNPs) that spread to the contralateral tympanum. Specifically, the yellow dotted circle represents the signal intensity relevant F-MNPs in the contralateral tympanum at the same sequence representing the axial and coronal scans, respectively. (**B**) Immunostaining of cochlear hair cells for each turn of ipsilateral and contralateral cochlear hair cells after intratympanic injection of F-MNPs. This figure represents rat number 2 in group 1.

**Figure 3:**
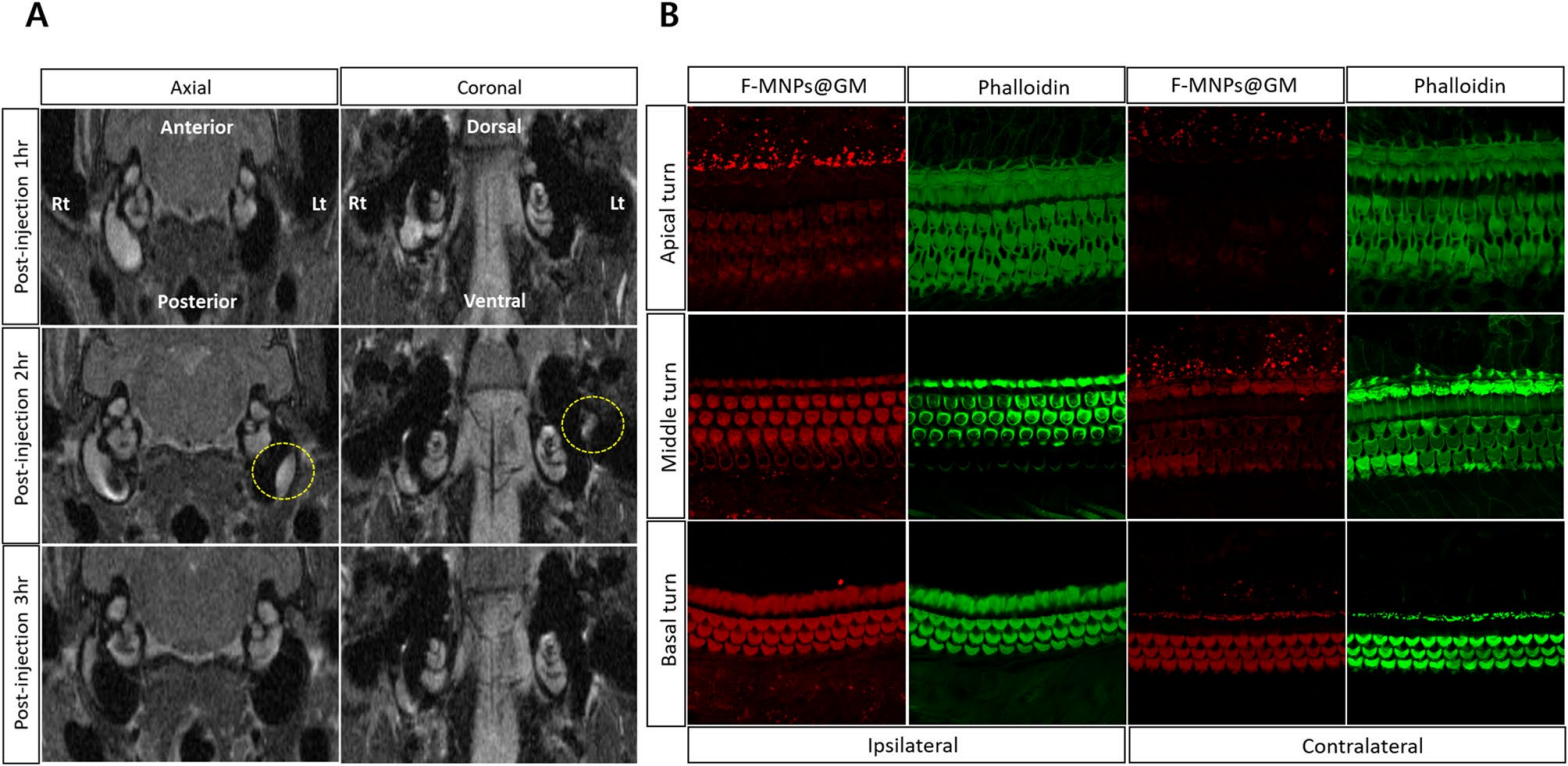
(**A**) Temporal visualization of intratympanically injected fluorescent magnetic nanoparticles (F-MNPs)-conjugated gentamicin (F-MNP@GM) spreading to the contralateral tympanum. Specifically, the yellow dotted circle represents the signal intensity relevant F-MNP@GM in the contralateral tympanum at the same sequence representing the axial and coronal scans, respectively. (**B**) Immunostaining of cochlear hair cells for each turn of ipsilateral and contralateral cochlear hair cells after intratympanic injection of F-MNP@GM. No F-MNP@GM-related fluorescence was depicted in the contralateral apical turn of the cochlea. This figure represents rat number 1 in group 2.

In contrast to our expectations, in the experimental groups (groups 1 and 2), there were no losses in signal intensity (negative contrast) or noticeable hypointense contrast of NPs on T2-weighted MRI of the middle or inner ear. In experimental group 1, heterogeneous enhancement was observed in the ipsilateral middle ear on images acquired 1 h after intratympanic injection of negatively charged F-MNPs. The heterogeneous signal intensity in the ipsilateral middle ear was detected in the contralateral middle ear on images acquired 2 h after administration. On sequential T2-weighted images, the extent of heterogeneous signal intensity increased in the contralateral ear and decreased in the ipsilateral ear over time (Fig. 2A). Consistent with the findings in experimental group 1, group 2 showed enhancement of F-MNP@GM in the contralateral middle ear on T2-weighted images acquired 1 h after intratympanic injection. On the images acquired 2 h later, F-MNP@GM gradually faded from the middle ear bilaterally (Fig. 3A). Additional images obtained whole T2-weighted sequences, which exhibit signal intensity associated with F-MNPs or F-MNP@GM, in the contralateral tympanum, are presented in Supplementary Fig. S2. Interestingly, contralateral spreading was exhibited in four of six (group 1 and group 2) rats following intratympanic injection of the F-MNPs or F-MNP@GM. That is, contralateral spreading was not observed in one animal each from group 1 and group 2, respectively. As expected, no filling effects or signal intensity were observed in the middle ear in group 3 (negative control).

### Visualization of F-MNPs and F-MNP-conjugated gentamicin

On cochlear whole-mount immunostaining with phalloidin, most outer hair cells (OHCs) showed an intact and organized configuration. In group 1, confocal microscopic examination demonstrated the cellular location of negatively charged F-MNPs in the ipsilateral and contralateral cochlear OHCs, especially in the basal turn (Fig. 2B). Bilateral distribution of NPs in the OHCs was found in two of four rats, corresponding to the features on MRI. The same findings were observed in experimental group 2, treated with F-MNP@GM (Fig. 3B). Furthermore, the distribution of F-MNP@GM was diluted from the base to the apex in the contralateral ear (Fig. 3B). As expected, no NP-relevant fluorescence was seen in group 3.

### Computational simulations: contralateral spreading of particles

**Figure 4:**
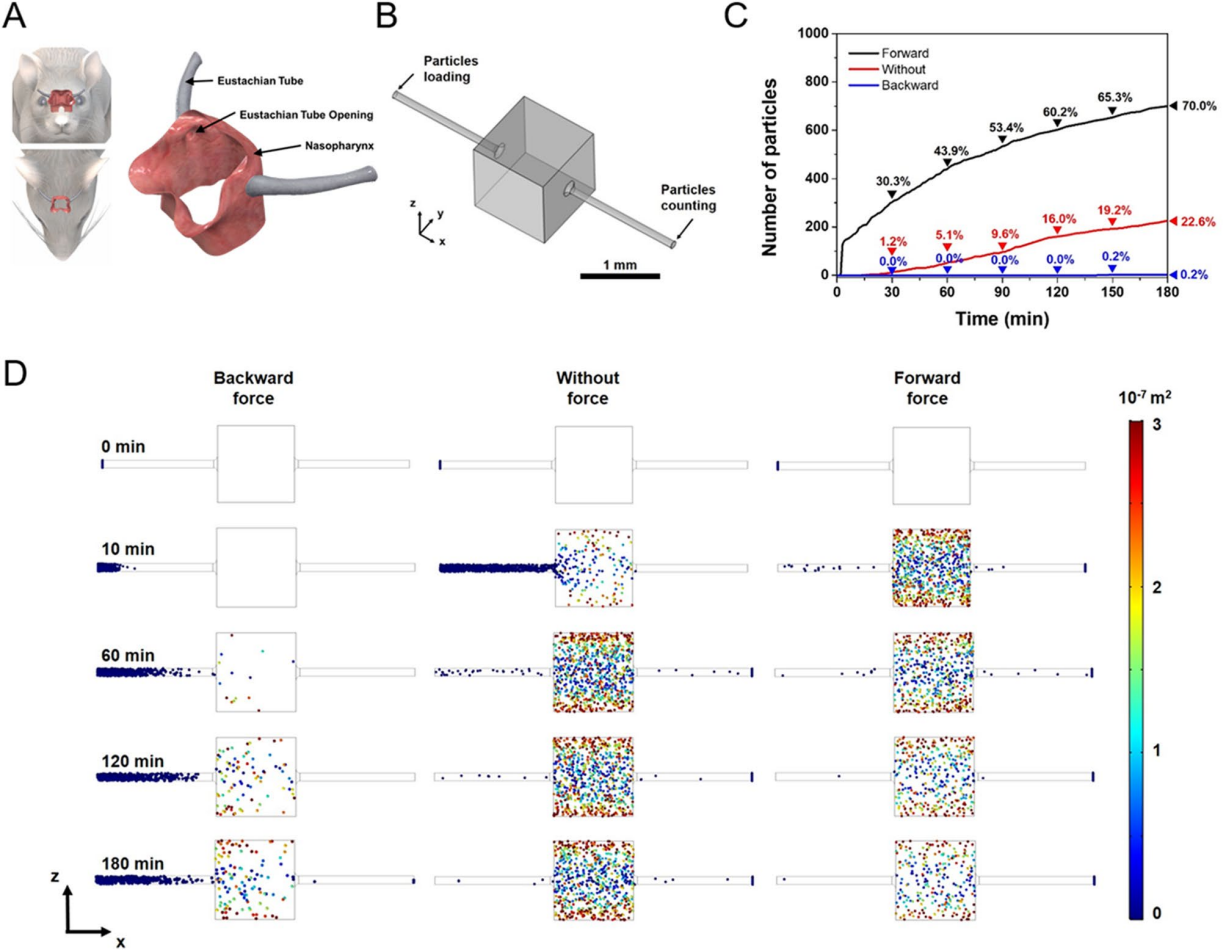
The simulation results of particle diffusion in the Eustachian tube. (**A**) The scheme of Eustachian tube anatomy of the rat. (**B**) Eustachian tube model for simulation geometry. (**C**) The number of particles arrived at the opposite side of the Eustachian tube by the time. (**D**) The simulation snapshots of particle diffusion in the Eustachian tube depending on time. For more clarify, the size of nanoparticles is denoted as a magnified scale. The color legend shows the position of the particle (y^2^ + z^2^) from the Eustachian tube axis (x-axis).

To further reinforce our findings, computational simulations were performed using COMSOL Multiphysics (see “Materials and methods” and Supplementary Information for details). The Eustachian tubes were modeled, and both sides were connected with the central cavity, which geometrically mimics the anatomy of the rats (Fig. 4A,B). First, the simulations were carried out with explicit the external force effect to describe the diffusion rate and direction of particles under the net force applied (Fig. 4C,D). The simulation snapshots indicate that the nanoparticles loaded at the ipsilateral side diffuse toward the contralateral side of the Eustachian tube (Fig. 4D). The number of particles that reached the end of the opposite side tube was counted with different force directions (Fig. 4C). The simulation results show that 22.6% of initially loaded nanoparticles have arrived at the other side tube without external force at 180 min after nanoparticle loading. When the forward force (+x direction) is applied toward the contralateral direction, the number of nanoparticles arrived at the opposite Eustachian tube is increased up to 70.0%. In contrast, when the backward force (−x direction) is applied against the contralateral direction, the number of nanoparticles arrived at the opposite side tube is decreased to 0.2%. Since the contralateral ear is located at the bottom side and some amount of time (e.g., 20–30 min) is given during the injection as proposed our transtympanic injection protocol, likely recapitulating clinical situation in humans, this computational simulation supports the conclusion that the possibility of contralateral spreading of particles by gravity force following intratympanic injection in a rat model. Moreover, the simulation results presented here imply that transtympanic approach-based drug delivery can be efficiently controlled by an additionally induced external force, e.g., magnetic gradient force. Indeed, magnetic nanoparticles have been used in our experiment, coupled with recent researches. Overall, modulating the direction or the strength of external forces, controlled drug delivery can be accomplished for customized the purpose.

In addition, the diffusion phenomenon caused by intrinsic particle properties was also clarified by simulations. To investigate the size-dependent diffusion effect, three different sizes of particles were modeled, i.e., 20 nm, 200 nm, and 2000 nm (Supplementary Fig. S3). At the 180 min after particle loading, 84.9% (20 nm), 24.2% (200 nm), and 0.0% (2000 nm) of initially loaded particles arrived at the opposite side of the Eustachian tube; thus the diffusion rate of particles is inversely proportional to the mass of particles. The surface charge of particles also affects the mobility of particles, which may be attributed to the additional electrostatic repulsion, resulting in an increase of diffusion rate. When the surface charge of particles is 0.1, 35.9% of particles arrived at the end of the Eustachian tube. At the charge of 1, 82.0% of particles were reached the contralateral side (Supplementary Fig. S4). Based on the simulation results, we can conclude that the induced external forces, e.g., gravity, magnetic force, can enhance the contralateral spreading of particles, which enables the guided drug delivery with a specific direction. Besides, the particle mobility and inter-particle collision are significant in natural diffusion; thus, the diffusion rate toward the contralateral side of the inner ear can be modulated by controlling the sizes and surface charges.

## Discussion

**Figure 5:**
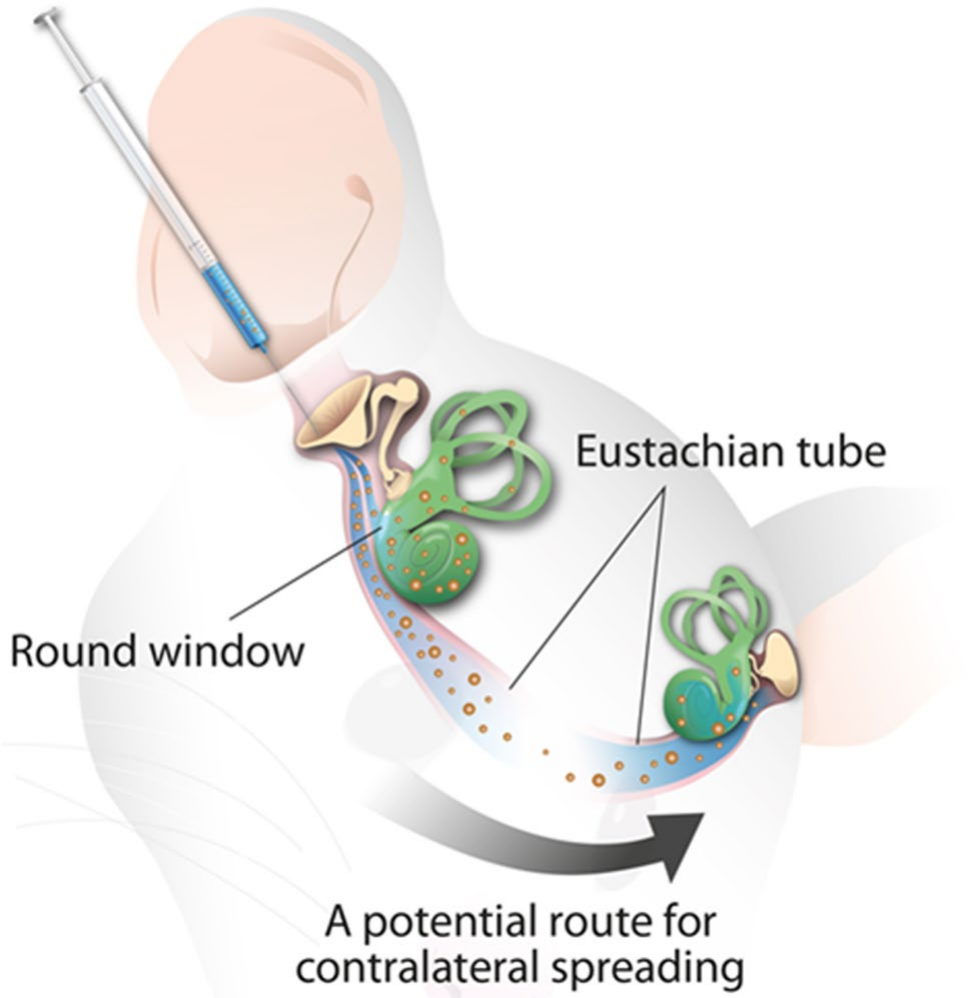
Hypothetical explanation of the contralateral spreading via the eustachian tube following the ipsilateral transtympanic injection of nanoparticle conjugated gentamycin.

This is the first study to suggest the Eustachian tube as a potential route for contralateral spreading following intratympanic injection of nanoparticle-conjugated gentamicin in a rat model. First, intratympanically injected materials that were observed in the ipsilateral middle ear were also detected in the contralateral middle ear over time. Second, only rats with the contralateral spreading on MRI showed fluorescence corresponding to NPs in the contralateral OHCs. Third, contralateral spreading was observed at a frequency of 66.7% (four of six) in the experimental groups. Finally, no radiological uptake or NP fluorescence was observed in the control group. As illustrated in Fig. 5, based on the detection of F-MNPs or F-MNP@GM ipsilaterally injected in the contralateral middle and inner ear temporally and spatially, our results suggest that the Eustachian tube via nasopharynx is the most likely route of contralateral spreading.

Considering gravity-dependent drains of middle ear fluid to the nasopharynx via the Eustachian tube, the extent of anatomical patency of the Eustachian tube may be a crucial determinant of contralateral spreading. Consistent with our findings, a previous *in-vivo* study evaluating the anatomy of the Eustachian tube in a rat model demonstrated that lack of attachment of muscle fibers in the ventral portion of the Eustachian tube could dilate the lumen, which could result in a higher rate of elimination^15^. Similarly, a previous study in a chinchilla model proposed that contralateral spreading may be attributable to transmission via the Eustachian tube due to the continuous opening of the Eustachian tube in this model^16^. A recent study revealed that the peak drug concentration and delivery time in the inner ear varied across intratympanic injections, suggesting that the Eustachian tube may have been responsible^17^. Although our experiments were performed under the same conditions, differential findings of contralateral spreading in terms of the presence, quantification, and clearance in the middle ear may be due to the extent of the anatomical patency of the Eustachian tube.

In addition, the physicochemical properties of materials may provide plausible explanations. In particular, the viscosity and osmolarity of agents may play a crucial role with respect to the clearance-to-diffusion ratio in the middle ear^18^. Several previous studies have indicated that adhesive agents were associated with a longer duration of contact with the round and oval windows^19,20^. Due to the lower viscosity of F-MNPs compared to gadolinium agents, they may be easily eliminated via the Eustachian tube, which in turn may increase the chance of contralateral spreading at a relatively high rate. In addition, higher osmolarity could cause higher diffusion in the inner ear fluid^18,21^. As F-MNPs have relatively lower osmolarity, they have a slower rate of absorption into the inner ear, and staying longer in the middle ear enhances their chances of clearance through the Eustachian tube.

It is worth noting that the F-MNP@GM showed dilution from the base to the apex on the contralateral and not on the ipsilateral ear when compared to F-MNPs alone. The F-MNPs and F-MNP@GM used in this study were approximately 338.5 nm and 434.3 nm in size, respectively (Table 2). The particle size would be a potential factor in determining the differential gradient of immunoreactivity between NPs in the contralateral side of the inner ear. Computational simulations suggest that controlling the particle sizes contribute to the diffusion rate toward the contralateral ear via the Eustachian tube, recapitulating the histological findings. In order to pursue this rationale, future in vivo studies with larger numbers of rats are warranted after adjusting confounders, such as physicochemical properties of materials and external force, that may affect particle diffusion.

**Table 2:** Particle size, zeta potential, and concentration of Fe ion of F-MNPs before and after gentamicin loading.

	Conjugation	Size (nm)	Zeta potential (mV)	Fe ion concentration (mM)	Combination
F-MNPs	NA	338.5 ± 4.7	− 22.5 ± 0.7	363	F-MNPs 200 mL + saline 1800 mL
F-MNP@GM	Covalent bonding (by EDC/NHS coupling)	434.3 ± 15.9	− 7.2 ± 0.5	453	F-MNPs (chemically) + gentamycin 40 mL + saline 1960 mL

*F-MNPs* fluorescent magnetic nanoparticles, *F-MNP@GM* F-MNP-conjugated gentamicin, *Rt* right, *Lt* left, *EDC/NHS* ethyl-3-(3-(dimethylamino)-propyl)carbodiimide/*N*-hydroxysuccinimide, *NA* not available.

Our results raise questions regarding the use of the contralateral ear as an appropriate control in animal studies investigating inner ear delivery. Based on our results, the contralateral ear should not be used as a control, as the relatively high chance of contralateral spreading renders the comparison unclear. Similarly, a recent study found that unilateral gentamicin injection led to immunolabeling of the vestibular efferent neurons bilaterally, and resulted in functional deterioration of the vestibular efferent neurons^22^. Agents with slow and sustained drug release, such as hydrogels, may be useful as alternatives in attenuation of contralateral spreading, based on their mechanism of prevention of drastic changes in middle ear capacity, as well as their potential stabilization of the permeability of the round window membrane^23^. Our animal study had clinical relevance, as transtympanic administration of gentamicin is widely performed in treatment of patients with intractable vertigo. A previous study showed that repeated transtympanic gentamicin injections led to bilateral vestibulopathy in some human cases, with the contralateral loss of cochlear or vestibular function in patients with unilateral vestibulopathy following transtympanic gentamicin injections^24^. Thus, our study results have provided crucial insight into the contraindication of the contralateral ear as a control ear, especially in animal studies that investigate inner ear delivery through the transtympanic approach.

Although this was the first study to suggest the Eustachian tube as a potential route of contralateral spreading, several limitations should be addressed with follow-up investigations. First, in addition to the Eustachian tube, investigations of other possible routes, such as the cochlear aqueduct or systemic vascular system, which may be associated with contralateral spreading, were not performed in this study. A previous study exhibited light immunostaining for gentamicin in the contralateral spiral ganglion cells with a lateral-to-medial gradient in the dorsal cochlear nucleus bordering the cochlear aqueduct^7^. Therefore, we cannot definitively conclude that the observed contralateral spreading was solely due to transmission via the Eustachian tube. Further studies using a control group with occlusion of the Eustachian tube and comparing systemic administration of F-MNP@GM are required. Second, the lack of quantification and stimulation of immunostaining NPs, and the relatively loose and insufficient timeline to evaluate the temporal distribution, may have limited our findings. Further, it remains elusive that whether or not the amounts of NPs for contralateral spreading based on computational simulation is compatible with the signal intensity found on MRI and immunostaining, even though our results suggest that the phenomenon of contralateral spreading could be doable within a similar time frame, regardless of the different methodologies. Lastly, we believe that the contralateral spreading via the Eustachian tube following intratympanic injection is not necessarily limited to NPs. As evidenced here, the diffusion phenomenon into the contralateral ear would occur depending on the physicochemical properties of substances, such as sizes and surface charges, as well as anatomical patency. Thus, we suggest that future studies with larger numbers of rats should be directed toward determining the contralateral spreading via the Eustachian tube after intratympanic injections using clinically applicable drugs, such as gentamicin and dexamethasone. The understanding will provide some insights into the field in relation to inner-ear drug delivery through the transtympanic approach.

## Conclusion

The Eustachian tube could be a route for the contralateral spreading of substances injected in the middle ear. Our results suggest that caution should be taken when using the contralateral ear as a control, especially in animal studies that investigate inner ear delivery through the transtympanic approach.

## Materials and methods

### Study design

All animal care and experimental procedures were approved by Seoul National University Bundang Hospital Institutional Animal Care and Use Committee (IACUC# 14-2017-003) and were conducted in accordance with internationally accepted guidelines. Intratympanic injections were administered as described previously^20^. In this study, the contralateral ear is located at the bottom side and some amount of time (e.g., 20–30 min) is given during the injection to expect absorption into the inner ear, likely recapitulating clinical situation in humans. However, all animals were placed in the prone position throughout MRI evaluation for 3 h to prevent position effects of leaning toward the left or right side. After administration of the intratympanic injection, whole T2-weighted MRI sequences corresponding to the regions of interest after 1, 2, and 3 h were taken and evaluated, along with immunostaining results of each turn of the cochlea. After image acquisition, each rat was immediately euthanized to harvest the membranous labyrinth of the cochlea from both ears.

### Preparation of F-MNP-conjugated gentamicin

Briefly, the F-MNPs were rinsed and dispersed in aqueous solution to 5 mg/mL. The F-MNPs (100 µL) were mixed with 20 mg of EDC [ethyl-3-(3-(dimethylamino)-propyl)carbodiimide] and 20 mg of NHS (*N-*hydroxysuccinimide) in 0.5 mL of MES buffer (pH 5.5) and incubated at 27 °C for 15 min. The F-MNPs were washed with MES buffer (pH 5.5) and mixed with gentamicin sulfate (5 mg) in 0.01 M PBS buffer (pH 7.4), after which the mixture was allowed to react at 27 °C for 2 h. The F-MNPs were finally washed with PBS containing 0.01% Tween 20 to remove unreacted molecules and stored at 4 °C until use.

### Characterization of F-MNP-conjugated gentamicin

Europium shell-coated F-MNPs synthesized using a modified sol–gel method were purchased from AMO LIFE SCIENCE Inc. (Amo-Mag; AMO LIFE SCIENCE Inc., Kimpo, Korea). F-MNP@GM was prepared by an EDC/NHS coupling reaction (further details are provided in the Supporting Information). The morphologies of the Eu-SiO_2_ shell-coated F-MNPs were characterized by high-resolution transmission electron microscopy (HR-TEM; JEM-2100F, JEOL, Tokyo, Japan). Photoluminescence (PL) measurements were obtained by fluorescence spectroscopy (F-7000; Hitachi, Tokyo, Japan). The particle size distribution and surface potential were measured by dynamic light scattering (DLS) analysis (Malvern Instruments, Malvern, Worcestershire, UK). The surface properties were monitored by FT-IR spectroscopy (FT-IR6300; JASCO, Tokyo, Japan).

### MRI protocol

A 9.4-T animal MRI scanner was used in combination with a volume coil (9.4 T/160AS; Agilent Technologies, Santa Clara, CA, USA)^20^. Prior to data acquisition, the rats were anesthetized with isoflurane (1.5% in oxygen) and placed inside the magnet in the prone position. The respiration rate and body temperature of the animals were monitored during MRI. After the acquisition of scout images, 2D T2-weighted axial and coronal images were sequentially obtained 1 h after intratympanic injection (fast spin-echo; repetition time, 3000 ms; effective echo time, 24 ms; flip angle, 90°/180°; echo train length, 4; field of view, 30 × 30 mm^2^; slice thickness, 0.3 mm; number of slices, 50 (axial) and 40 (coronal); matrix size, 256 × 256; bandwidth, 100 kHz; number of signal averages, 8). SPION causes a loss of signal intensity (“negative contrast”) on T2-weighted images^25^. To visualize NPs on MRI, we obtained whole sequences of images associated with the regions of interest, including the middle and inner ear, and analyzed them using the M-view system (Marosis, Seoul, Korea).

### Tissue preparation

As described in our previous report, after decapitation under anesthesia, the cochleae from both ears were immersion-fixed with 4% paraformaldehyde (pH 7.4)^26^. Via surface preparation, the membranous labyrinth was dissected under a microscope and fixed with 4% paraformaldehyde. Cochlear whole-mount preparations were produced as follows: (1) the tissues were soaked in 0.3% Triton X-100 blocking solution for 1 h; (2) the fixed tissues were labeled with Alexa 488-conjugated phalloidin for 1 h, washed, and fixed with 4% paraformaldehyde; and (3) the tissues were mounted on slides with anti-fade fluorescence mounting medium (Vector Laboratories, Burlington, ON, Canada). The immunolabeled samples were imaged using a Zeiss 710 confocal microscope (Carl Zeiss MicroImaging, Oberkochen, Germany) at a uniform magnification of 63×. A wavelength of 647 nm was used for NP excitation.

### Computational simulation

The finite element method (FEM) simulation (COMSOL Multiphysics 5.2a, Boston, US) was used for estimating particle diffusion. In size-dependent calculation, different radius, mass, collision cross-section of particles was used for three different sizes of particles (Supplementary Table S1). For charge and external force-dependent calculation, the size of nanoparticles is fixed as 200 nm and the coulomb interactions were counted in addition to the diffusion to consider the electrostatic inter-particle interactions of charged particles. The length of the Eustachian tube is 1.5 mm long with a radius of 0.05 mm. The nasopharynx was modeled as a cubic with the length of one side is 1 mm. The volume of Nasopharynx is 1 mm^3^.

## Supplementary information

Supplementary Table S1.

Supplementary Figure S1.

Supplementary Figure S2.

Supplementary Figure S3.

Supplementary Figure S4.

## Data Availability

Data for all submitted results is available.
