# Supplementary material for: Contralateral spreading of substances following intratympanic nanoparticle-conjugated gentamicin injection in a rat model
Source: Sci Rep. 2020 Oct 29;10:18636. doi: 10.1038/s41598-020-75725-y (PMC7596480; doi:10.1038/s41598-020-75725-y)
Supplement: Supplementary file 1 — Supplementary Table S1. [file 41598_2020_75725_MOESM1_ESM.pdf]

Contralateral spreading of substances via Eustachian tube following intratympanic nanoparticle-conjugated gentamicin injection in a rat model

Sang-Yeon Lee[1,6], Jeonghyo Kim[2], Sangjin Oh[2], Gaon Jung[1], Ki-Jae Jeong[3], Van Tan Tran[2,4], Dajeong Hwang[2], SungIl Kim[5], Jae-Jin Song[1], Myung-Whan Suh[6], Jaebeom Lee[2*] and Ja-Won Koo[1*]

Table S1. Simulation parameters in computational stimulations

Size (nm)	Radius (m)	Mass (kg)	Collision cross-section (m^2)
20	1E-8	2.17E-20	1.26E-15
200	1E-7	2.17E-17	1.26E-13
2000	1E-6	2.17E-14	1.26E-11
