# Supplementary material for: Contralateral spreading of substances following intratympanic nanoparticle-conjugated gentamicin injection in a rat model
Source: Sci Rep. 2020 Oct 29;10:18636. doi: 10.1038/s41598-020-75725-y (PMC7596480; doi:10.1038/s41598-020-75725-y)

Contralateral spreading of substances via Eustachian tube following intratympanic nanoparticle-conjugated gentamicin injection in a rat model

Sang-Yeon Lee[1,6], Jeonghyo Kim[2], Sangjin Oh[2], Gaon Jung[1], Ki-Jae Jeong[3], Van Tan Tran[2,4], Dajeong Hwang[2], SungIl Kim[5], Jae-Jin Song[1], Myung-Whan Suh[6], Jaebeom Lee[2*] and Ja-Won Koo[1*]

Figure S1. (A) Scanning electron microscopy (SEM) images of fluorescent magnetic nanoparticles (F-MNPs) before and after Eu^{3+}-doped SiO_2 shell coating. (B) Hydrodynamic diameter and zeta potential of F-MNPs during the process of drug loading. (C) Bright-field and photoluminescence microscopic images of 1D alignment of F-MNPs.

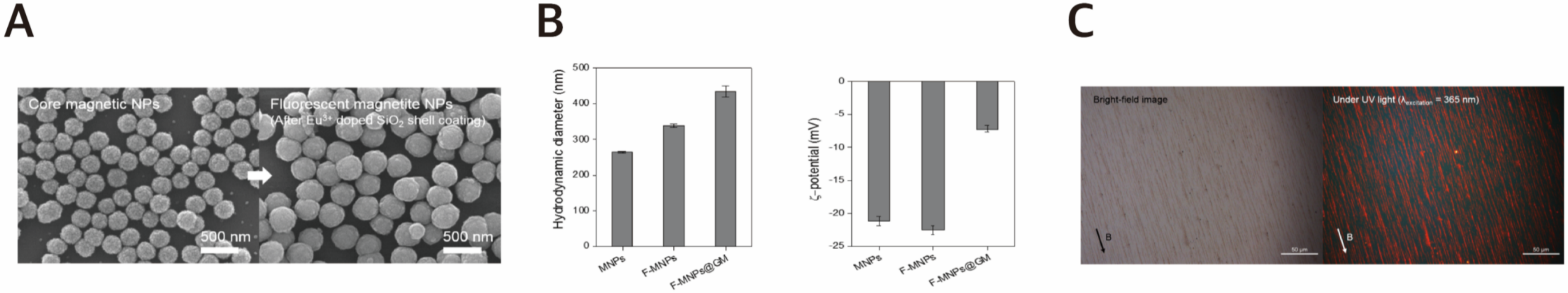

Supplement: Supplementary file 2 — Supplementary Figure S1. [file 41598_2020_75725_MOESM2_ESM.pdf]
