# Supplementary material for: Contralateral spreading of substances following intratympanic nanoparticle-conjugated gentamicin injection in a rat model
Source: Sci Rep. 2020 Oct 29;10:18636. doi: 10.1038/s41598-020-75725-y (PMC7596480; doi:10.1038/s41598-020-75725-y)

Contralateral spreading of substances via Eustachian tube following intratympanic nanoparticle-conjugated gentamicin injection in a rat model

Sang-Yeon Lee[1,6], Jeonghyo Kim[2], Sangjin Oh[2], Gaon Jung[1], Ki-Jae Jeong[3], Van Tan Tran[2,4], Dajeong Hwang[2], SungIl Kim[5], Jae-Jin Song[1], Myung-Whan Suh[6], Jaebeom Lee[2*] and Ja-Won Koo[1*]

Figure S2. (A) Temporal visualization of transtympanically injected fluorescent magnetic nanoparticles (F-MNPs) that spread to the contralateral middle ear in whole T2-weighted sequences, (B)Temporal visualization of transtympanically injected fluorescent magnetic nanoparticles (F-MNPs)-conjugated gentamicin (F-MNP@GM) that spread to the contralateral middle ear in whole T2-weighted sequences.

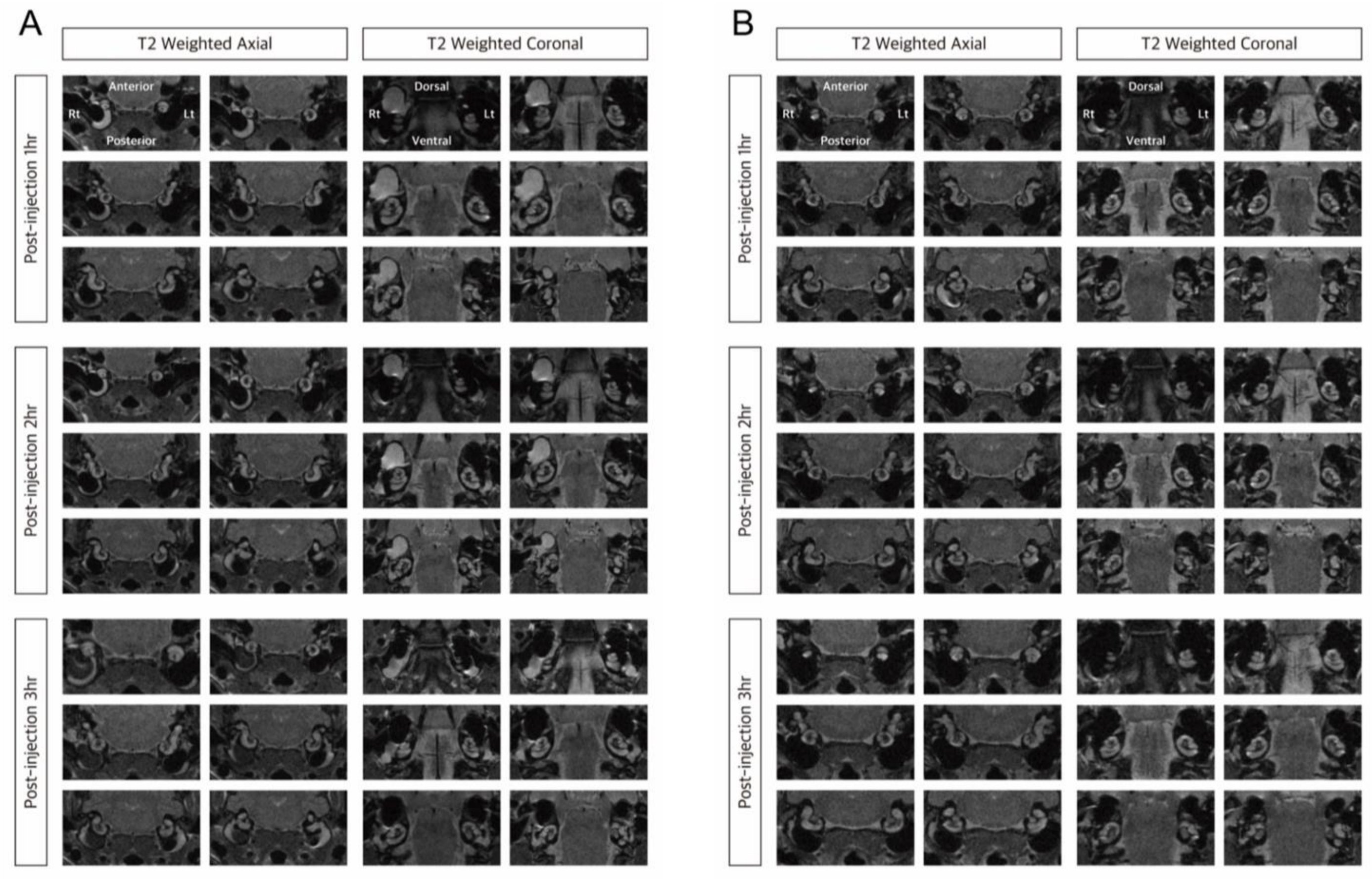

Supplement: Supplementary file 3 — Supplementary Figure S2. [file 41598_2020_75725_MOESM3_ESM.pdf]
