# Supplementary material for: Contralateral spreading of substances following intratympanic nanoparticle-conjugated gentamicin injection in a rat model
Source: Sci Rep. 2020 Oct 29;10:18636. doi: 10.1038/s41598-020-75725-y (PMC7596480; doi:10.1038/s41598-020-75725-y)

Contralateral spreading of substances following intratympanic nanoparticle-conjugated gentamicin injection in a rat model

Sang-Yeon Lee[1,6], Jeonghyo Kim[2], Sangjin Oh[2], Gaon Jung[1], Ki-Jae Jeong[3], Van Tan Tran[2,4], Dajeong Hwang[2], SungIl Kim[5], Jae-Jin Song[1], Myung-Whan Suh[6], Jaebeom Lee[2*] and Ja-Won Koo[1*]

Figure S4. The simulation results of particle charge-dependent diffusion in the Eustachian tube. (A) The simulation snapshots of particle diffusion in the Eustachian tube depending on time. (B) The number of particles arrived at the opposite side of the Eustachian tube by the time.

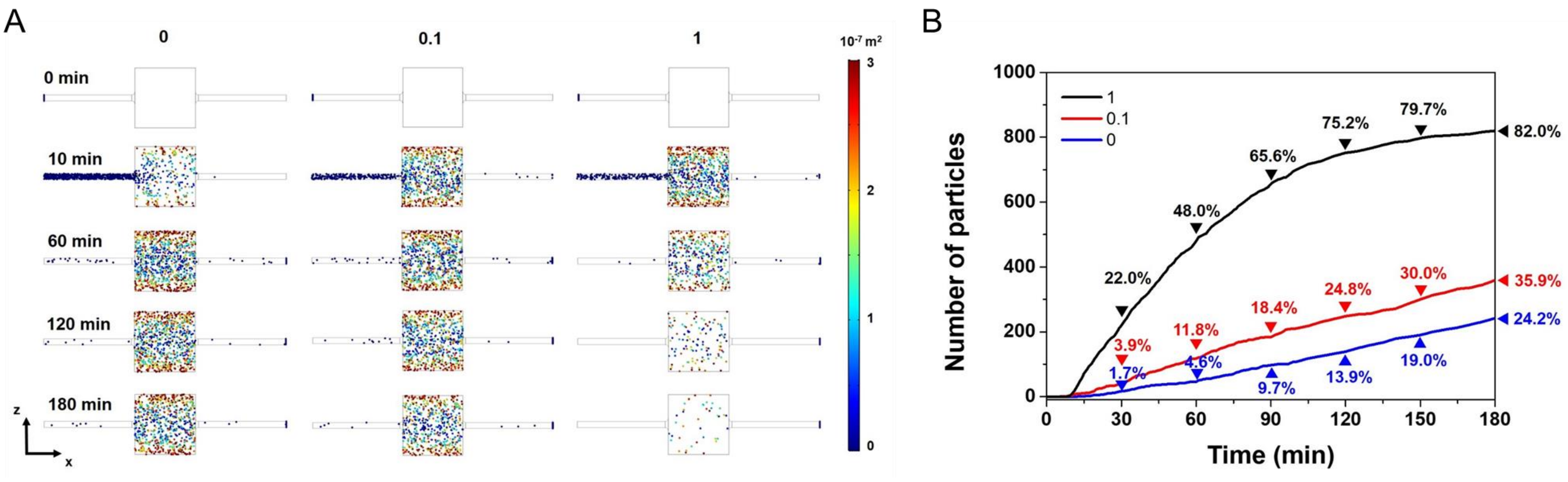

Supplement: Supplementary file 5 — Supplementary Figure S4. [file 41598_2020_75725_MOESM5_ESM.pdf]
